# Metastatic Melanoma Presenting As Small Bowel Obstruction

**DOI:** 10.7759/cureus.113827

**Published:** 2026-08-02

**Authors:** Amanda Ruci, Sarah M Chan, Carolyn K Kan, Chris Fagel, Jetrina Maque

**Affiliations:** 1 Internal Medicine, University of California, Los Angeles, Los Angeles, USA; 2 Internal Medicine, University of California Los Angeles, Los Angeles, USA; 3 Internal Medicine, West Los Angeles VA, Los Angeles, USA

**Keywords:** gi metastasis, malignant small bowel obstruction, metastatic cutaneous melanoma, metastatic melanoma, small bowel obstruction

## Abstract

Metastatic melanoma to the gastrointestinal (GI) system is a known, though overall rare, phenomenon. Typically, symptomatic presentations from GI metastases are uncommon and often nonspecific, contributing to delayed diagnosis. We present the case of a male patient with a history of cutaneous melanoma treated with wide local excision one and a half years earlier, who presents to the emergency department with presyncope, acute nausea, vomiting, and melena. Initial workup revealed a five-point hemoglobin drop. Computed tomography (CT) of the abdomen and pelvis showed a high-grade small bowel obstruction with a soft tissue mass at the transition point. The patient underwent a diagnostic laparoscopy, small bowel resection, and lymph node dissection. Histopathology and immunohistochemical markers confirmed the diagnosis of malignant melanoma, which, along with the patient's history of cutaneous melanoma, favored a metastatic etiology. This case demonstrates the need to maintain a high index of suspicion for metastatic melanoma in patients with a prior history of cutaneous melanoma, especially for those presenting with bowel obstruction or obscure GI symptoms. Surgical resection is the mainstay of therapy and can be both diagnostic and therapeutic.

## Introduction

Melanoma is the fifth most common type of cancer in the United States, with population-based data showing that 4.7% of patients present with distant metastases at the time of diagnosis, while the majority (77%) have localized disease [1]. While early stage (IA-IB) cutaneous melanoma is often curable with surgical excision, its metastatic potential is well recognized, and distant recurrence can occur years to decades after initial treatment [2]. The risk of recurrence and distant metastasis is closely associated with the clinicopathologic characteristics of the primary melanoma, including Breslow thickness, ulceration, mitotic activity, lymphovascular invasion, and sentinel lymph node status. These features provide important prognostic information and help guide surveillance strategies following surgical resection. Melanoma commonly metastasizes to the lungs, liver, brain, and bones. However, gastrointestinal (GI) metastases from melanoma are underrecognized and have been identified in up to 60% of patients with melanoma at autopsy [3].

The small bowel is the most frequent site of GI involvement (80%), accounting for the majority of cases [4-6]. Antemortem diagnosis of small bowel melanoma metastasis is often delayed, as patients are usually asymptomatic or present with nonspecific symptoms, such as abdominal pain, nausea, vomiting, weight loss, or occult GI bleeding [4]. Acute presentations of GI involvement, such as small bowel obstruction (SBO), intussusception, or hemorrhage, are less common but represent life-threatening surgical emergencies and may serve as the first clinical manifestation of metastatic disease [7].

SBO as the initial presentation of metastatic melanoma is particularly uncommon and diagnostically challenging [8]. The interval between initial melanoma diagnosis and GI recurrence can span years, and patients may not volunteer their cutaneous oncologic history as pertinent medical history when being evaluated for seemingly unrelated GI symptoms [9]. A critical diagnostic challenge is distinguishing metastatic cutaneous melanoma from primary gastrointestinal melanoma, two different disease processes which are histologically and immunohistochemically indistinguishable [3,10,11]. Thus, clinical context and dermatologic history are of paramount importance for diagnosis [3].

We present a case of metastatic melanoma to the small bowel manifesting as a high-grade SBO in a patient with a prior history of excised cutaneous melanoma. This report aims to raise clinical awareness of this rare but critical presentation, review relevant literature, and discuss the approach to management of melanoma metastasis to the GI tract.

## Case presentation

A 62-year-old male with a history of stage IA (pT1aN0M0) right flank cutaneous melanoma (Breslow thickness 0.7mm, without ulceration) treated with wide local excision without sentinel node biopsy one and a half years ago, hypertension, hyperlipidemia, and basal cell carcinoma presents to the emergency department with acute-onset nausea, vomiting and presyncope. The patient reported having onset of symptoms less than 24 hours prior to presentation, along with a two-week duration of black stools. He denied aspirin, anticoagulant, or other antiplatelet use.

**Figure 1:**
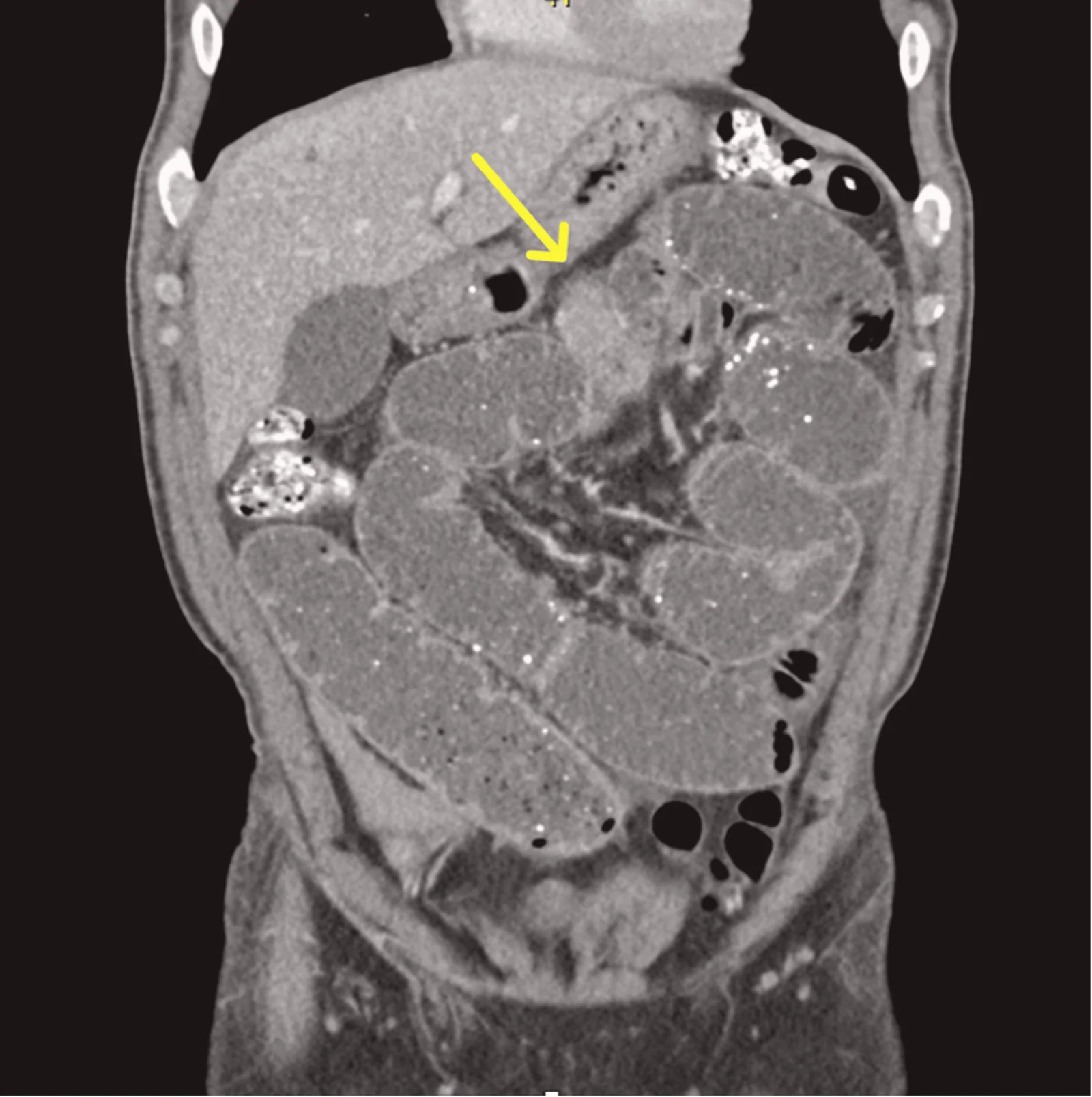
Coronal CT abdomen and pelvis demonstrating a soft tissue mass at the transition point (yellow arrow), leading to small bowel obstruction.

**Figure 2:**
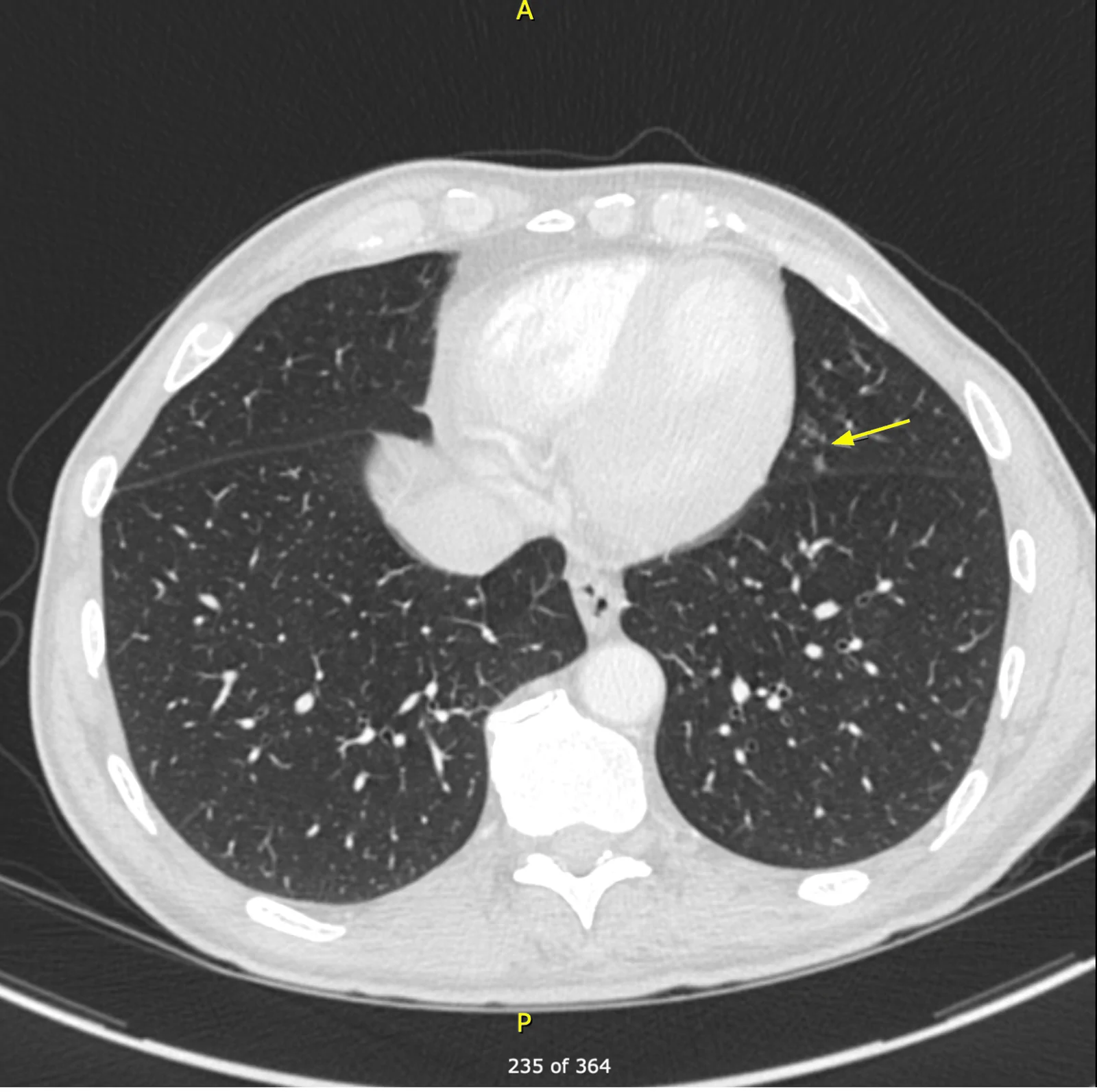
Axial CT chest demonstrating few clustered centrilobular solid micronodules in the inferior lingula (yellow arrow), without evidence of metastasis disease elsewhere in the chest.

**Figure 3:**
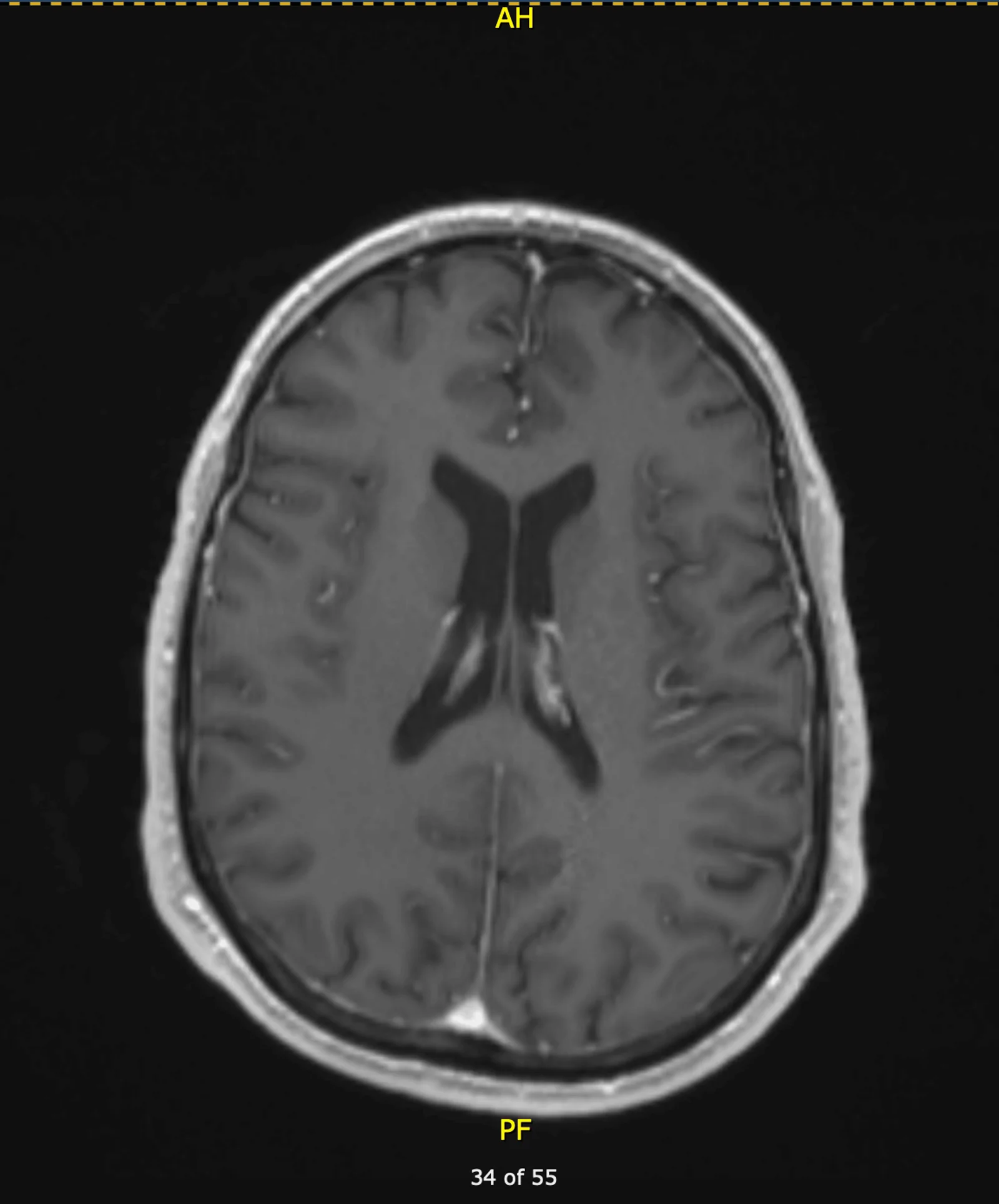
Axial post-contrast T1-weighted MRI brain demonstrating no acute intracranial hemorrhage, mass effect, midline shift, or intracranial metastasis.

In the emergency department, vitals signs were notable for blood pressure of 86/63 mm Hg, heart rate 106 beats per minute, temperature 98.1°F, and oxygen saturation of 99% on room air. Physical exam was notable for a soft, nondistended, nontender abdomen without palpable masses. Initial bloodwork revealed a hemoglobin of 9.1 g/dL, decreased from a baseline of 14.0 g/dL (Table 1). His basic metabolic panel and liver function tests were within normal limits (Table 1). He was given one liter of intravenous fluid followed by maintenance fluids and pantoprazole 80 mg intravenously followed by 40 mg two times daily, along with antiemetics, with improvement in blood pressure to 115/74 mm Hg and heart rate to 95 beats per minute. No blood products were given as he did not have gross evidence of gastrointestinal bleeding. Computed tomography (CT) of the abdomen and pelvis (Figure 1) showed a high-grade small bowel obstruction (SBO) with a transition point in the mid-pelvis, unable to exclude underlying mass lesion, along with precaval/mesenteric lymph nodes. The patient subsequently underwent CT enterography, which again showed high-grade SBO, though this time notable for a soft tissue mass at the transition point, concerning for malignancy. Tumor markers [carcinoembryonic antigen (CEA), carbohydrate antigen (CA) 19-9] were negative. CT chest revealed a few clustered solid micronodules suggestive of infectious or inflammatory etiology, without definitive evidence of pulmonary metastases (Figure 2). Magnetic resonance imaging (MRI) of the brain showed no intracranial metastatic disease (Figure 3).

**Table 1:** Initial laboratory findings on presentation AST: aspartate aminotransferase; ALT: alanine aminotransferase.

Laboratory Test	Patient Value	Reference Range
White Blood Cell Count (x10E3/uL)	12.32	4.16-9.95
Hemoglobin (g/dL)	9.1	13.5-17.1
Hematocrit (%)	33.0	38.5-52.0
Platelet Count (x10E3/uL)	518	143-398
Sodium (mmol/L)	138	135-146
Potassium (mmol/L)	4.6	3.6-5.3
Chloride (mmol/L)	99	96-106
Total CO2 (mmol/L)	25	23-30
Urea Nitrogen (mg/dL)	19	7-22
Creatinine (mg/dL)	1.20	0.6-1.3
Glucose (mg/dL)	113	65-99
Calcium (mg/dL)	10.0	8.6-10.4
AST (U/L)	20	13-62
ALT (U/L)	29	8-70
Total Bilirubin (mg/dL)	0.6	0.1-1.2
Alkaline Phosphatase (U/L)	69	37-133
Albumin (g/dL)	4.2	3.9-5.0

**Figure 4:**
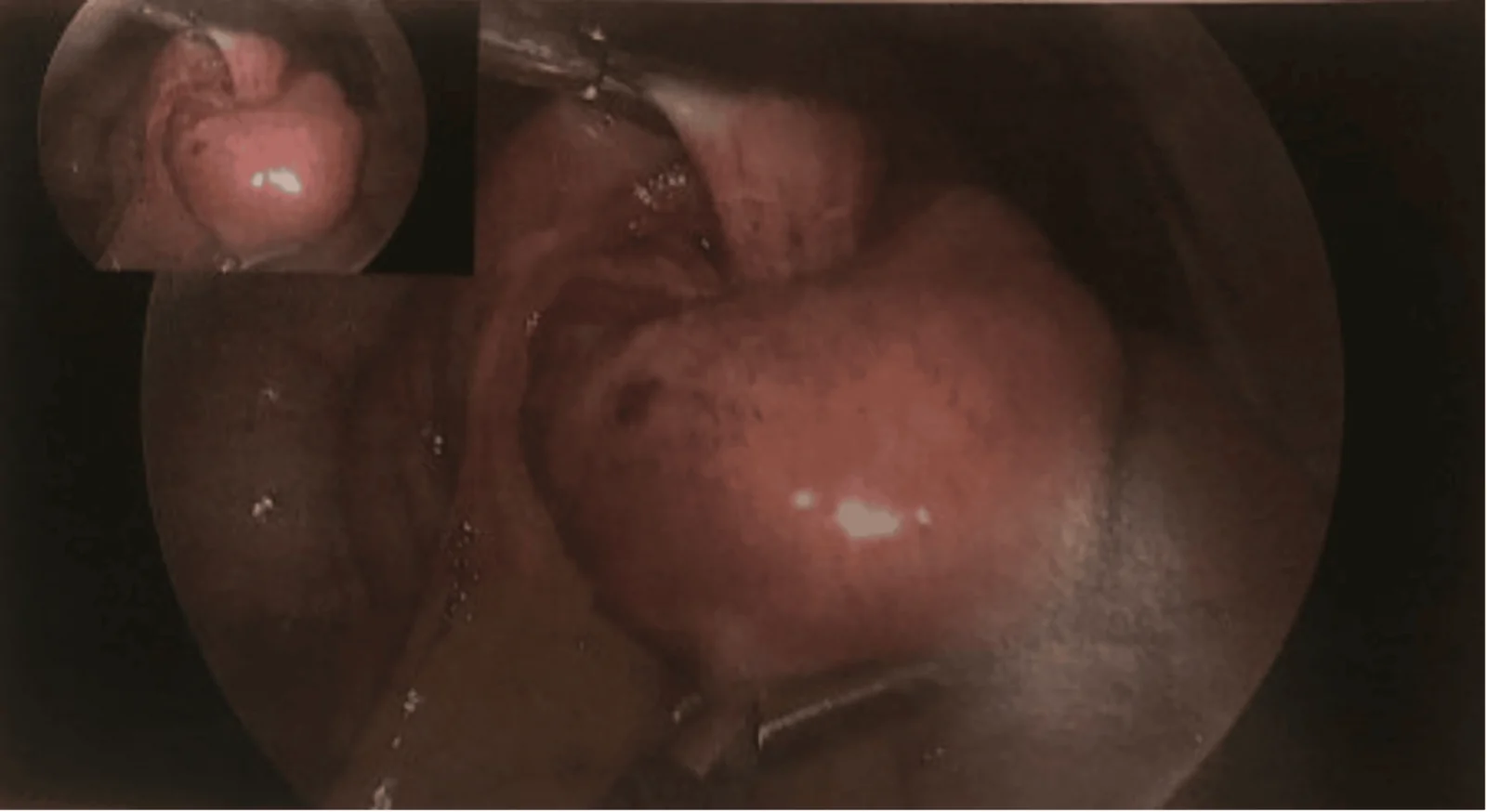
Intraoperative laparoscopic image demonstrating the soft tissue mass to the small bowel transition point prior to resection.

General surgery was consulted and performed a diagnostic laparoscopy with removal of a 4.2-centimeter (cm) soft tissue mass from the transition point (Figure 4) on hospital day five. Small bowel resection with primary anastomosis was performed, with 18 total lymph nodes removed. His post-operative course was unremarkable, and he was discharged on hospital day eight.

Pathology of the mass confirmed malignant melanoma. Histopathology demonstrated tumor involvement of the muscularis propria and ulceration of overlying bowel luminal epithelium. The surgical margins were negative for the tumor. Metastatic melanoma was identified in one of the 18 lymph nodes examined, with a deposit measuring 0.7 cm. Lymphovascular invasion was present in the primary bowel lesion.

Immunohistochemistry demonstrated strong positivity for SOX10, diffuse positivity for melanoma antigen recognized by T-cells (MART1), and positivity for human melanoma black (HMB45) and preferentially expressed antigen in melanoma (PRAME), supporting the diagnosis of melanoma (Table 2). Molecular profiling via pan-cancer solid tumor next-generation sequencing (NGS) panel identified a V-Raf murine sarcoma viral oncogene homolog B (BRAF) V600K mutation with a variant allele frequency of 40%, wherein BRAF positivity has known applications for targeted systemic therapies. The patient was then placed on induction therapy of nivolumab and ipilimumab (PD-1 inhibitor and CTLA-4 inhibitor, respectively) complicated by hemophagocytic lymphohistiocytosis (HLH). HLH resolved following discontinuation of nivolumab and ipilimumab and treatment with high-dose steroids. He was subsequently transitioned to maintenance therapy of nivolumab for a planned total duration of two years, with CT scans of the chest, abdomen, and pelvis every four to six months and skin exams every three to six months. His recent CT abdomen and pelvis and MRI brain showed no evidence of metastatic disease.

**Table 2:** Immunohistochemistry report CAM 5.2: anti-cytokeratin mouse monoclonal antibody; SOX10: sex-determining region Y-related high mobility group-box 10; MART1: melanoma antigen recognized by T-cells; HMB45: human melanoma black; PRAME: preferentially expressed antigen in melanoma.

Antibody/Probe	Result
Pankeratin	Negative
CAM 5.2	Negative
SOX10	Positive, strong diffuse
MART1	Positive, diffuse
HMB45	Positive
PRAME	Positive

## Discussion

Melanoma to the GI tract has been previously described in the literature as a possible metastatic site, yet it remains diagnostically challenging due to its variable and protracted clinical course [4]. The small bowel is the most common site of gastrointestinal involvement, occurring in about 80% of all GI metastases in autopsy-based studies [4-6]. However, clinically symptomatic presentations are far less common, and SBO as the initial manifestation of metastatic melanoma is especially rare [3]. Estimates of GI metastases being detected before death range from 1.5% to 4.4% [3].

Melanoma can exhibit late and distant recurrences [12]. This case of metastatic cutaneous melanoma occurring one and a half years after resection despite appropriate surveillance for stage IA disease (patient was seen every three to six months by dermatology for comprehensive skin exam) supports the need for ongoing oncologic surveillance in patients with melanoma. As evidenced by a review of 1,623 patients with abdominal visceral melanoma metastases, patients found to have gastrointestinal metastasis incidentally on routine surveillance imaging had improved overall survival time when compared to patients who presented with nonspecific gastrointestinal symptoms (39 months versus 19 months, p <0.001) [13]. Late recurrences have been documented more than 10 to 15 years after initial diagnosis, particularly with deeper primary lesions [12,14].

The pathophysiology of SBO in the setting of metastatic melanoma most commonly involves intraluminal polypoid or submucosal deposits [3]. These lesions may lead to partial or complete obstruction, intussusception, or volvulus. Hematogenous spread is the predominant mechanism of metastasis, favoring highly vascular regions, such as the submucosal layer of the small bowel [15].

An important diagnostic consideration is the distinction between cutaneous melanoma with metastasis to the GI tract versus primary GI melanoma. Primary GI melanoma is exceedingly rare and is a diagnosis of exclusion [10,11]. While melanocytes are not native to the GI mucosa, their presence has been hypothesized to arise from neural crest cell migration during embryogenesis or from amine precursor uptake and decarboxylation (APUD) cells within the gut wall [10]. Metastatic cutaneous melanoma, by contrast, accounts for the vast majority of melanoma found in the GI tract [10]. Histologically and immunohistochemically, the two malignancy types are indistinguishable, both staining positively for S-100, HMB-45, Melan-A, and SOX10, making a thorough clinical history and full-body skin exam essential for a diagnostic workup [3,16]. The presence of a prior primary cutaneous melanoma, as in our patient, strongly favors a metastatic etiology [17]. In any patient with GI melanoma without a known primary melanoma, a comprehensive dermatologic evaluation, including dermoscopy and full body skin exam, is warranted before a diagnosis of primary GI melanoma can be considered [18]. 

Surgical resection remains the mainstay of treatment for metastatic cutaneous melanoma involving the GI tract, both for symptomatic improvement and for potential survival benefit in appropriate patients [13]. Studies have demonstrated improved overall survival in patients undergoing complete resection of isolated GI metastases compared to those managed non-operatively, with median survival reported between 12 and 24 months post-resection in favorable cases [13]. Specifically, in appropriate candidates (patients with potentially resectable disease in whom complete, curative resection could be achieved), it is recommended for patients to undergo both surgical resection and systemic immunotherapy as the backbone of treatment [18].

The surgical approach to both primary GI melanoma and metastatic cutaneous melanoma involving the GI tract is similar, with the main goals being obstruction relief, hemorrhagic control, and complete resection when feasible [9]. Distinguishing between primary GI melanoma and metastatic cutaneous melanoma is important, as it has significant implications for prognosis and treatment selection. Primary GI melanoma harbors distinct molecular features, including a lower rate of BRAF V600 mutations, higher prevalence of KIT mutations, and lower tumor mutational burden [10]. These differences can result in significantly reduced response rates to immune checkpoint inhibitors and BRAF/ mitogen-activated protein kinase (MEK)-targeted therapy and are associated with substantially worse prognosis [10]. Primary GI melanomas harboring KIT mutations may be responsive to therapy with KIT inhibitors (imatinib). However, metastatic cutaneous melanoma to the GI tract is more likely to harbor BRAF mutations, allowing patients to benefit from broader therapeutic options including BRAF/MEK inhibitors [10]. In this case, the patient had a BRAF V600 mutation with an allele frequency of 40%, a molecular profile more consistent with a cutaneous origin, and was placed on immune checkpoint inhibitor therapy [10,11]. Additionally, the patient underwent diagnostic laparoscopy with resection of the obstructing mass and small bowel, along with regional lymph node dissection, allowing for simultaneous pathologic diagnosis and therapeutic intervention.

This case highlights several important factors in approaching the diagnosis. First, a comprehensive oncologic history must be obtained in all patients presenting with bowel obstruction, particularly those with histories of skin cancers given the risk of metastatic disease. Second, negative standard tumor markers should not prevent pursuing tissue diagnosis when imaging findings are suspicious of malignancy. Third, multidisciplinary coordination between emergency medicine, internal medicine, surgery, pathology, dermatology, and oncology is essential for timely diagnosis and appropriate management.

## Conclusions

Metastatic melanoma presenting as small bowel obstruction remains a rare clinical entity that should be included in the differential diagnosis, especially in patients with a history of cutaneous melanoma. GI metastases are typically asymptomatic. When symptoms do occur, they are often nonspecific, and the potentially long latency period between primary diagnosis and metastatic recurrence further delays diagnosis. Additionally, standard tumor markers are unreliable in these cases. Cross-sectional imaging is imperative to identifying obstructing lesions and to guiding surgical planning. Molecular findings, together with the clinical and pathologic context, help identify patients’ overall malignancy origin. 

Surgical resection remains the mainstay of treatment for GI melanoma metastases, both for symptomatic improvement and for potential survival benefit in appropriate patients. Postoperative systemic therapy with immune checkpoint inhibitors or targeted agents should be considered following multidisciplinary coordination and molecular characterization of the resected tumor.

This case demonstrates the importance of ongoing oncologic monitoring in patients with a history of melanoma, even with appropriate excision and treatment. This case also reinforces the importance of considering an underlying malignancy in patients presenting with SBO. Prompt surgical excision and comprehensive pathologic workup are critical in making the diagnosis and initiating appropriate interventions.

## References

[1] Joshi UM, Kashani-Sabet M, Kirkwood JM (2025). Cutaneous melanoma: a review. JAMA.

[2] Osella-Abate S, Ribero S, Sanlorenzo M (2015). Risk factors related to late metastases in 1,372 melanoma patients disease free more than 10 years. Int J Cancer.

[3] Lens M, Bataille V, Krivokapic Z (2009). Melanoma of the small intestine. Lancet Oncol.

[4] Yee EJ, Thielen ON, Truong R (2025). Metastatic melanoma to the small bowel and colon: a systematic review of the global experience and institutional cohort analysis detailing a rare clinical entity. J Surg Oncol.

[5] Othman AE, Eigentler TK, Bier G (2017). Imaging of gastrointestinal melanoma metastases: correlation with surgery and histopathology of resected specimen. Eur Radiol.

[6] Ollila DW (2006). Complete metastasectomy in patients with stage IV metastatic melanoma. Lancet Oncol.

[7] Cibulas MA, Carrillo EH, Lee SK, Rosenthal AA (2022). Jejuno-jejunal intussusception secondary to recurrent/metastatic melanoma. Am Surg.

[8] Malissen N, Farvacque G, Duconseil P (2021). Surgery of small bowel melanoma metastases in the era of efficient medical therapies: a retrospective cohort study. Melanoma Res.

[9] La Selva D, Kozarek RA, Dorer RK, Rocha FG, Gluck M (2020). Primary and metastatic melanoma of the GI tract: clinical presentation, endoscopic findings, and patient outcomes. Surg Endosc.

[10] De Nardi P, Guida S, Damiano G (2026). Primary melanoma of the gastrointestinal tract. World J Gastroenterol.

[11] Xie J, Dai G, Wu Y (2022). Primary colonic melanoma: a rare entity. World J Surg Oncol.

[12] Faries MB, Steen S, Ye X, Sim M, Morton DL (2013). Late recurrence in melanoma: clinical implications of lost dormancy. J Am Coll Surg.

[13] Deutsch GB, Flaherty DC, Kirchoff DD (2017). Association of surgical treatment, systemic therapy, and survival in patients with abdominal visceral melanoma metastases, 1965-2014: relevance of surgical cure in the era of modern systemic therapy. JAMA Surg.

[14] Reschke R, Dumann K, Ziemer M (2022). Risk stratification and clinical characteristics of patients with late recurrence of melanoma (>10 years). J Clin Med.

[15] Amersi FF, Terando AM, Goto Y (2008). Activation of CCR9/CCL25 in cutaneous melanoma mediates preferential metastasis to the small intestine. Clin Cancer Res.

[16] Sanchez AA, Wu TT, Prieto VG, Rashid A, Hamilton SR, Wang H (2008). Comparison of primary and metastatic malignant melanoma of the esophagus: clinicopathologic review of 10 cases. Arch Pathol Lab Med.

[17] Ohsie SJ, Sarantopoulos GP, Cochran AJ, Binder SW (2008). Immunohistochemical characteristics of melanoma. J Cutan Pathol.

[18] Ribero S, Pampena R, Bataille V (2016). Unknown primary melanoma: worldwide survey on clinical management. Dermatology.

